# Supplementary material for: On the use of in-silico simulations to support experimental design: A case study in microbial inactivation of foods
Source: PLoS One. 2019 Aug 27;14(8):e0220683. doi: 10.1371/journal.pone.0220683 (PMC6711534; doi:10.1371/journal.pone.0220683)
Supplement: Attachment — Submitted filename: reviewer_comments_june_A2_J2.docx [file pone.0220683.s003.docx]

**MS Number PONE-D-19-14978**

Title: On the use of in-silico simulations to support experimental design: a case study in microbial inactivation of foods

Authors: Alberto Garre, Jose Lucas Peñalver-Soto, Arturo Esnoz, Asunción Iguaz, Pablo S. Fernández, Jose A. Egea

**Journal: PLOS ONE**

**ITEMIZED LIST OF REPLY TO REVIEWERS COMMENTS**

We would like to thank the reviewers and editor for their insightful comments on the paper, as they led us to improve its overall quality. Please find below our replies to the comments, including details about the corresponding changes incorporated in the new revised version of the manuscript.

**Comments from the reviewers:**

**Reviewer #1:**

Summary:
The authors discuss the use of OED for identifying the Bigelow model for thermal microbial inactivation. Specifically, the manuscript focusses on the selection of the sampling times and the type of temperature profile.

Major comments:
- The statement on lines 88 and 89 is either not really correct or the authors did not clearly state what they meant. Since OED has been applied in predictive microbiology for over 20 years, it definitely falls already in the category of “currently available techniques”. As stated in the article, by simply calculating the inverse of the FIM for a proposed experimental design, it is already possible to have an idea of the precision that will be obtained on the parameter estimates (and this is just the underlying principle of OED). Therefore, currently available techniques can definitely provide such information.

- In general, the final section of the introduction (lines 88-100) should more clearly state the novelty of this research. Is it just providing information on optimal sampling schemes? Or is it the underlying method for determining such sampling schemes? Try to state more clearly what knowledge was already available and what is novel about this work with respect to the available research.

We acknowledge both reviewer’s comments and agree in the fact that the novelty of this research was not correctly described in the original manuscript. We have rewritten the final part of the introduction addressing the two major comments.

“…The superiority of D-optimal designs with respect to uniform designs has been already demonstrated in several works (e.g. (Balsa-Canto et al., 2008; Garre et al., 2018b; Stamati et al., 2016).

“Despite the advances in OED, there is still high uncertainty in experimental design in the context of predictive microbiology. The most common goal of this type of experiments is the calculation of parameter estimates with a precision above a minimum (standard deviation of the model parameters). To date, there is not a clear methodology to estimate this. Therefore, some parameters of the design (e.g. the number of sampling points) is selected based on previous experience. Hence, there is a high risk that the number of sampling points is excessive, leading to unnecessary experimental work, or too low, which would require posterior repetitions of the experiment. In this work, we explore the application of numerical techniques to reduce this uncertainty. We propose two complementary methodologies, the first one based on the properties of the FIM and the second one based on Monte Carlo simulations. Although they have been applied in previous studies to compare between different designs, here we describe their mathematical basis and illustrate how they can be used to aid experimental design, reducing the risk of designs with excessive or too few sampling points. For this, we analyze a case study related to dynamic microbial inactivation. Nevertheless, the applicability of these methods is not restricted to this case and could, in principle, be applied to any problem in the context of predictive microbiology. “

- The penalty function that is used (lines 153-162), appears to provide a weighting that is very arbitrary. Why don’t the authors apply constraints on the sampling times to be optimised? Using a set of linear inequality constraints could lead to a much less arbitrary solution to the same problem.

We have used in this work the same penalization scheme that was used in Garre et al. (2018), where we explored different ways to describe the constraint. We agree that for most “classical” optimization algorithms linear inequality constraints are usually the best way to define this type of constraints. However, the optimization problem to be solved in the FIM-based OED requires a global optimization method which overcomes non-convexity issues. For that reason, we chose MEIGO which, as a metaheuristic, follows a very different strategy for the optimization. For this algorithm, linear inequality constraints were way less efficient (number of function evaluations) than the penalty function.

Nevertheless, we believe that a comparison of different ways to define the constraint is out of the scope of this research work. Note that our goal is the identification of experimental designs that are the most informative while, at the same time, being feasible from an experimental point of view. The way we have formulated the problem enables to calculate this in a reasonable time (some minutes) in a laptop (Windows 10, 1 core with 8 GB of RAM). More efficient formulations may exist for this particular problem, but exploring them would bring little benefit to the objective of this particular research work.

- For the results presented in Section 3.1, the sampling points of the OED designs should be illustrated and discussed. Use, e.g., a relative frequency bar chart to illustrate all sample points over the Monte Carlo simulation. These charts can be represented as three subfigures, corresponding with those of Figure 1. It should be possible to link these sampling points with the sensitivity equations in Figure 3.

We acknowledge the reviewer’s comment and agree that this is a relevant point that was not addressed in the original manuscript. As suggested, we have added a new subsection to the R&D section where the OEDs are described (section 3.1). It includes a new plot (Figure 2 in the new version). The new section now reads (L201-243):

## 3.1 Description of the Optimal Experiment Designs identified

Figure 2 gives a qualitative description of the designs identified as optimal for each one of the profiles analyzed (Figure 1), as well as on how they vary when the number of samples is increased. The x-axis represents the sample space (the duration of the experiment) and the height of the bars the number of samples that are located at a given location. The total number of sample points is represented by the colour of the bar (see legend in Figure 2). The bars corresponding to different number of points are stacked on top of each other, so the total height of the bar is a representation of the use of a time point across a different number of samples.

For every profile analyzed, some areas of the sampling space are the most informative and the algorithm tends to locate samples in that location. As expected, the most informative design pattern depends on the shape of the thermal profile. These differences between profiles can be justified based on the local sensitivity functions of each profile, shown in Figure 3. For profile A, samples are located in close to the end of the treatment, at approximately 56 and 60 minutes (Figure 2A). As illustrated in Figure 3A, $t=56$ corresponds to a minimum of the sensitivity function with respect to the z-value and $t=60$ to a supremum of both sensitivity functions. The algorithm is able to identify these areas and locates the sampling points in a configuration that satisfies the constraint related to the minimum distance between samples. For profile A, due to the large duration of the experiment (60 min) with respect to the minimum time between samples (3s), the restriction can be easily fulfilled. Hence, the frequency plot (Figure 2A) shows a large area without any samples between the two informative areas.

**Figure 2:** Frequency bar plot illustrating the OED calculated for the three profiles analyzed (A, B and C). The colour of the bar indicates the total number of sampling points.

**Figure 3:** Scaled local sensitivity functions of profile A (A), B (B) and C (C) with respect to the D-value (**-**) and the z-value (**--**).

Although profile C has a shape resembling the one of profile A, there are several differences that affect the optimal design pattern. Whereas the optimum design pattern for profile A distributes the samples in a balanced manner between *t = 56* and *t = 60*, for profile C the end of the experiment is favoured. This is due to the fact that the sensitivity functions (especially the one of the z-value) grow quickly with temperature for temperatures about the reference one. The maximum temperature in profile C is 65°C, 5°C higher than the one reached in profile A. Consequently, the minimum of the sensitivity function with respect to the z-value is less relevant in profile C than in profile A. Furthermore, the duration of profile C is much lower than the one of profile A (2 min vs 60 min). Therefore, the constraint related to the minimum distance between sampling points is much harder to fulfil and the design is more spread-out.

Profile B has a shape very different from those of profiles A and C. Hence, the optimal design pattern for this profile (Figure 2B) is very different to the one of the other two profiles. Several samples are located close to the middle of the treatment (*t = 28* and *t = 32*). Both points correspond, respectively, to a minimum and a maximum of the sensitivity function corresponding to the z-value (Figure 3B). Besides this area, samples are located at the end of the experiment, where the sensitivity with respect of the D-value reaches its highest value.

- It is not really clear to me what the advantage is of the Monte Carlo method proposed in this publication. Why would you not just use the approximation of the variance-covariance matrix based on the inverse of the FIM of your design to estimate the uncertainty on the model parameters? What is the added value of this Monte Carlo simulation?

The reviewer raises an interesting concern that was not properly addressed in the 1^st^ version of the manuscript. The FIM has several shortcomings as an estimator of the variance-covariance matrix (C). First, according to the Cramer-Rao inequality, the FIM is a lower bound of the C. Moreover, the FIM does not take into account non-linearities in the model (10.3389/fbioe.2019.00122). Furthermore, the formulation of the FIM used in this work (as well as in most scientific works) is based on the hypothesis that the model is perfect and that errors are normal.

The article has been expanded discussing this:

(L329): “On the other hand, the FIM has several shortcomings as an estimator of the variance-covariance matrix (*C*) of the model parameters. According to the Cramer-Rao inequality, the FIM is a bound of *C* under several hypotheses that disregard possible non-linearity in the model (Krausch et al., 2019). Moreover, the calculation of the FIM and the local sensitivities can be complicated when errors are non-normally distributed, for instance when residuals are heteroscedastic or when they do not follow a normal distribution (e.g. a Poisson distribution). Also, local sensitivity functions can be hard to calculate when the parameter of interest is the variance (e.g. in studies such as (Aryani et al., 2015)). Monte Carlo simulations are more flexible than the procedure based on the FIM are can be applied in such cases with little complexity.”

(L458): “Also, more complex hypotheses that are hard to implement in the FIM (e.g. heteroscedasticity of the residuals) can be implemented in the Monte Carlo simulations assess their impact. “

Minor comments:

- Line 21: Remove “)”.

Thank you for the comment. Corrected.

- Figures 1 - 3: Use a line width of minimum 2 for the curves.

The comment is acknowledged. The plots have been remade accordingly.

- Line 211: calculate -> calculating.

Thank you for the comment. Corrected.

- Line 311: Takes what into account? Complete this sentence.

We acknowledge the reviewer’s comment. The sentence has been rewritten as

“OED takes into account the amount of information when the number of sampling points is increased by one. On the other hand, uniform designs simply place the new point according to a uniform partition of the sampling space.”

- Line 334-335: Use a capital letter for “Van Derlinden”.

Thank you for the comment. Corrected.

- Line 345: smallest -> smaller.

Thank you for the comment. Corrected.

- Simulation data should be made available.

We agree with the reviewer’s comment. In the revised version, we have added the code used for the simulations as supplementary material.

**Reviewer #2**

The manuscript shows an interesting case study of microbial inactivation in foods with in-silico simulation analysis to support the suggestion of the two complementary methodologies to predict the parameter precision for a given experimental design. The manuscript is sound, but some major concerns are listed, and minor corrections are suggested below.

Major:
- Lines 115-121: The three profiles proposed have very different heating rates which impact on microbial inactivation. What assumptions were made to propose these temperature profiles? Can authors propose other temperature profiles, based on some thermal treatment of real food or other more realistic profiles for the case study (e.g. with residence time)? This is a concerning limitation issue, as suggested by own authors (lines 196-198) “Although previous studies have proposed algorithms to select optimal profiles, this example will be limited to the study of the three inactivation profiles shown in Figure 1”.

In lines 197-207, the parameters considered on the case study are described. They include the number of sampling points, their location and a selection between 3 different temperature profiles. We are analysing designs with between 3 and 20 sampling points. This means that we are comparing 108 different possible experimental designs (18 number of samples, uniform/OED, 3 profiles). Including the shape of the profile as an additional factor would increase very much the number of designs to be analysed without adding a significant information to the study.

Regarding the effect of the heating rates, this is a very interesting point. Their impact on the heating rates is still an active research field (e.g. 10.1016/j.ijfoodmicro.2017.11.023; 10.1016/j.foodcont.2012.05.042). Therefore, it is not possible to include this factor in this type of analysis. This hypothesis was already described in the original version of the manuscript (L122-129: “Although there is experimental evidence indicating otherwise…”)

- Lines 144-145: Did authors test FIM criterions other than D-optimal? Why was D-optimal chosen? Advantages/disadvantages of that criterion against others in the context of the study (model and assumptions) should be presented.

We acknowledge the reviewer’s comment. We opted for using the D-criterion because it had been successfully used in similar problems. Being a relevant comment, we believe that comparison among optimality criterions is out of the scope of this research. The text has been modified discussing this point:

” Other criteria have been suggested to identify OEDs. A popular alternative is the E-criterion, which tries to minimize the maximum error (the model parameter with the highest error) (Balsa-Canto et al., 2008). Nevertheless, the D-criterion is preferred in some circumstances met in the case study presented here (Balsa-Canto et al., 2010) “

- Lines 229-232: “One hundred Monte Carlo simulations have been performed, considering ten sampling points for each experimental design (OED and uniform) for each temperature profile. Simulations have been repeated with a higher number of simulations without observing differences in the results (not shown)”. Why the simulation tests started from ten sampling points? Kinetic inactivation experiments often have less than ten sampling points due to practical experimental issues. Furthermore, experiments should be simulated from less than ten sampling points in order to identify differences in the results (since no differences were reported with ten or more sampling points).

We acknowledge the reviewer comment. The actual meaning of the paragraph (that we had made sure that the Monte Carlo algorithm had converged) was not clear in the original manuscript. We have rewritten that paragraph clarifying this topic.

“As described, the precision of the parameter estimates have been analysed using Monte Carlo simulations. In order to ensure the convergence of the algorithm, calculations have been repeated for different number of Monte Carlo simulations. Increasing their number beyond 100 simulations had no impact on the results (not shown), so the results obtained with 100 Monte Carlo simulations are reported.”

We have added a new subsection to the R&D section where the OEDs are described (section 3.1). This also helps to clarify the reviewer concern.

- Lines 249-251: “Therefore, the shape of the temperature profile should be taken into account when designing experiments for characterization of microbial inactivation under dynamic conditions”. Did authors try to design optimal temperature profiles before or together with optimal sampling points?

This point is, on its core, the same as MC#1. The shape of the temperature profile as a factor would very much increase the computational time without adding essential information to the results, which show that OED outperforms uniform designs in every case.

Minor:
- Lines 22-24. Present some reference about “scarce contributions”.

Although we appreciate the reviewer’s comment, the guidelines for PlosONE discourage the use of references in the abstract. In the introduction, this point is further discussed including references.

- Lines 78-81: FIM to quantify information in OED has been applied before Lehmann and Casella (1998).

We acknowledge the comment. The last part of the introduction has been rewritten clearly stating the novelty of the work.

- Line 120: 0.5 ºC/min and 10 ºC/min (and instead comma).

We appreciate the reviewer’s comment. Corrected.

- Line 147: The “equation” (2) is incomplete (equal to?).

We appreciate the reviewer’s comment. However, that is the standard way of writing an optimization problem (https://en.wikipedia.org/wiki/Optimization_problem).

- Lines 183-226: Many information is about method and should be presented in appropriate section. Unnecessary repeated information can be removed. Results effectively start to be shown at line 231.

We understand the reviewer comment. However, it must be understood that in this work analysis methods are not just a tool to analyse the data and reach conclusions. This research proposes the application of numerical methods to aid in the experimental design before any sampling point is taken. Hence, we believe that the discussion regarding how these can be actually applied and their limitations belongs to the R&D section, rather than to M&M. Future studies where this methodology is applied should describe it in M&M, but we believe that they belong to R&D in this work.

- Lines 242-243: “most of the inactivation occurs in a short time at the end of the experiment”. In Profile B most of the inactivation occurs at the middle of the experiment. In the end of the experiment, almost no inactivation occurs (due to the low temperature), as can be seen in Figure 1.

The reviewer’s comment is acknowledged. However, we are referring just to profile C in this sentence. It has been rewritten for clarification:

“…because most of the inactivation for profile C occurs in a short time (half a minute) at the end of the experiment”

- Lines 298-318: Authors discussed about loss of information in uniform design measured by the inverse of the FIM determinant. In Figure 2C, there is an unexpected loss of information of OED experiment when adding from 16 to 17 points. How authors can explain this fact?

The results shown in former Figure 2 (now Figure 4 after creating a new one and renumber the rest as suggested by Reviewer #1) require the application of several numerical methods (OED, sensitivity functions, determinant of the FIM, optimization) that introduce a numerical error (truncation and round-up) in the result. This is introducing some “noise” in the results shown in (former) Figure 2. Nonetheless, the OED remains much more informative than the uniform design. Moreover, according to the results of this investigation, increasing the number of sampling points for this profile beyond 12 samples is simply unreasonable.

The text has been modified clarifying these points:

“…This impact is progressively diminished as the number of sampling points is increased. Indeed, it plateaus for profile C for more than 12 sampling points. This is caused by the constraint regarding the minimum distance between sampling points, that does not enable to increase the amount of information. Therefore, there is a limit in the amount of information that can be extracted for a thermal profile, when a restriction is included to limit the minimum distance between sampling points. For a number of samples beyond this limit, the inverse of the determinant of the FIM may slightly increase due to the numerical error involved in the calculations. Nonetheless, from the results in Figure 2C, it is unreasonable to increase the number of samples beyond 12 for profile C.”

- Lines 374-375: “(3) that increasing the number of points in a uniform design does not ensure a higher precision”, as well as in some OED (e.g. profile C).

This point is discussed in the previous minor correction.

- Lines 471-472: Garre et al. 2017b reference is incomplete.

We acknowledge the reviewer’s comment. It has been completed.

- Figure 3: The elements of the figure should have complete description in the caption. Information about A and B, red and green curves.

We understand that this information is already present in the legend: “Scaled local sensitivity functions of profile A (A) and B (B) with respect to the D-value (**-**) and the z-value (**--**).” “A” shows the sensitivity functions for profile A and “B” for profile B. The red curve is the sensitivity with respect to the D-value and green, dashed curve with respect to z.

- Results of Monte Carlo simulations to D and z values could be used to presented and assess additional information.

 We acknowledge the comment. The code for the simulations has been included as supplementary information, so that anyone can reproduce the calculations.
