# Supplementary material for: On the use of in-silico simulations to support experimental design: A case study in microbial inactivation of foods
Source: PLoS One. 2019 Aug 27;14(8):e0220683. doi: 10.1371/journal.pone.0220683 (PMC6711534; doi:10.1371/journal.pone.0220683)
Supplement: Attachment — Submitted filename: reviewer_comments_July_A1_J1.docx [file pone.0220683.s004.docx]

**MS Number PONE-D-19-14978R1**

Title: On the use of in-silico simulations to support experimental design: a case study in microbial inactivation of foods

Authors: Alberto Garre, Jose Lucas Peñalver-Soto, Arturo Esnoz, Asunción Iguaz, Pablo S. Fernández, Jose A. Egea

**Journal: PLOS ONE**

**ITEMIZED LIST OF REPLY TO REVIEWER COMMENTS**

We would like to thank the reviewer and editor for their insightful comments on the paper, as they led us to improve its overall quality. Please find below our replies to the comments, including details about the corresponding changes incorporated in the new revised version of the manuscript.

**Comments from the reviewer:**

**Reviewer #3:**

The manuscript focusses on the use of Optimal Experimental Design (OED) to identify the most informative sampling schedule for the calibration of the Bigelow model for thermal microbial inactivation.
Despite an overall improvement can be detected in the second version of the manuscript, some of the concerns highlighted by previous reviewers have not been adequately addressed by the authors.

Major comments
- [Lines 105- 120]: The authors should clarify what to they mean by ‘there is still high uncertainty’ in the experimental designs used in predictive microbiology. Indeed the common tendency to set aspects of the experimental scheme adopted for model calibration based on past experience/technical limitations of the experimental system/acquisition platform applies to the proposed investigation (where only the effect of the number and location sampling times is considered, albeit not optimised). The authors should clearly state the reason for which they focus only on sampling times, as they could have investigated the effect of other aspects (e.g. the thermal perturbation profile). In addition, the use of scalar functions of the FIM to optimise experimental schemes is now routine. In this context, the actual contribution of this manuscript should be emphasised.

We acknowledge the reviewer’s comment. By “high uncertainty” we refer to a very common question in any microbiology laboratory: “how many sampling points do I need?” This can refer, for instance, to the number of sampling points taken in an inactivation experiment for characterizing the microbial response. This is usually decided based on experience. As a result, there is a high risk of designing experiments with too many points (resulting in additional experimental work) or with too few (requiring a repetition of the experiment). In this article, we propose the application of numerical techniques as a science-based approach to aid in experimental design.

In this research we optimize the position of sampling times. We apply the methodology for OED we previously developed (Garre et al., 2018b) to identify optimal sampling schemes (Section 2.2; Figure 2). Including the shape of the thermal profile as a parameter of the experimental design is an interesting exercise from the point of view of the experimental design that was studied in a previous research work (van Derlinden et al., 2010). However, we have some concerns regarding that approach from a biological point of view. It has been demonstrated that the shape of the thermal profile (i.e. the heating rate) can affect the microbial response. If the heating is slow, a physiological response can be triggered in the cells that increases their stress resistance (Corradini and Peleg, 2009; Dolan et al., 2013; Garre et al., 2018c, 2018a; Hassani et al., 2007, 2006; Stasiewicz et al., 2008; Valdramidis et al., 2007). Fast heating profiles can also influence the bacterial response to stress (de Jong et al., 2012; de Jonge, 2019; Huertas et al., 2016). The model-based approach for OED applied here is based on the hypothesis that the design does not affect the validity of the model (i.e. of the model parameters). We understand that including the shape of the temperature profile as a variable in the optimization problem collides with this hypothesis, due to the experimental evidence which indicates that it affects the microbial response. Nevertheless, the effect of heating rates on microbial inactivation is still an active research topic. A detailed discussion of this matter would take the focus out of the main research question addressed in the article, so it has not been included in the manuscript.

We disagree with the claim that OED is common practice in predictive microbiology. Although it is more commonly used in other fields (e.g. systems biology), uniform sampling schemes are still the default approach for describing microbial growth and/or inactivation under non-isothermal conditions (Conesa et al., 2009; Corradini and Peleg, 2004; Franco-Vega et al., 2015; Haberbeck et al., 2013; Huang, 2013; Huertas et al., 2015; Mattick et al., 2001; Ros-Chumillas et al., 2015; Valdramidis et al., 2008). We firmly believe that OEDs have several advantages with respect to uniform designs, but they must be illustrated with case studies before they are broadly accepted by the community (especially scientists without a strong background in statistics). This article serves that purpose, showing the drawbacks of uniform designs: lower precision for the same experimental effort, and the fact that they not ensure that increasing the number of sampling points reduces parameter uncertainty.

The last paragraph of the introduction has been largely rewritten including these remarks:

“

The model parameters of models used in predictive microbiology usually have a biological meaning. For instance, the D-value describes the treatment time required to reduce the microbial count a 90% (Bigelow, 1921). Model parameters estimated under certain conditions are commonly used to, for example, infer the effectiveness of a treatment (Maté et al., 2016; Ros-Chumillas et al., 2017). Therefore, in many situations, the objective of experiments designed in the context of predictive microbiology is not prediction but the estimation of model parameter with enough precisión (standard deviation) that enables accurate inference. Despite the advances in OED, there are still some open questions when it comes to designing such experiments. For instance, the number of sampling points is commonly decided based on previous experience. As a result, there is a high risk that the number of sampling points is excessive, leading to unnecessary experimental work, or too low, which would require posterior repetitions of the experiment. In this work, we explore the application of numerical techniques to reduce this uncertainty. We propose two complementary methodologies, the first one based on the properties of the FIM and the second one based on Monte Carlo simulations. Although both methods are usually applied to compare between different designs, here we illustrate how they can be used to aid in the decision process during the first stages of the experimental design. We describe their mathematical basis and illustrate how they can provide valuable information that may reduce the uncertainty of the experimental design (e.g. in the selection of the number of sampling points). For this, we analyze a case study related to dynamic microbial inactivation. Nevertheless, the applicability of these methods is not restricted to this case and could, in principle, be applied to any problem in the context of predictive microbiology.

“

- The adopted formulation of the penalty function to set a constraint on the sampling frequency requires clarification. I understand that a similar formulation was adopted in a previous publication by the same authors, but i) hybrid solvers can cope with linear inequality constraints in FIM-based OED; ii) the optimisation cost cannot be too high in the considered case (1D model with 2 parameters).

We appreciate the reviewer’s suggestion. In a previous investigation (Garre et al., 2018b), we tried different formulations of the optimization problem and we found that the addition of the penalty function provided feasible solutions which could not be improved by applying a final local search with a local method (i.e., interior point method) which handles non-linear constraints. In this research, we have used a global optimization method, as recommended by different authors to address this type of problem (e.g., Balsa-Canto et al. 2008). We also tried another global optimization method handling inequality constraints, the Improved Stochastic Ranking Evolution Strategy (ISRES), implemented in the nloptr R package (<https://cran.r-project.org/web/packages/nloptr/index.html>) and after much longer runs than those performed with MEIGO and the mentioned penalty function, the obtained solutions were worse than those presented in our work. We also used the Rsolnp method (<https://cran.r-project.org/web/packages/Rsolnp/index.html>), which is a general non-linear optimization method using augmented Lagrange multipliers and, again, the results did not outperformed those obtained with the proposed formulation/method. The best solutions were provided by the enhanced scatter search method implemented in MEIGO using a final local refinement with a direct search method based on hill climbing (DHC). The package bioOED referenced in our paper allows users to use MEIGO with its different options and local solvers to solve OED problems in predictive microbiology.

We are aware that we do not have a mathematical prove that the obtained solutions are optimal, but we obtain better solutions than other alternatives, we use the recommended type of solve for this type of problem, and we believe that the possible difference between the provided solutions and the mathematically optimal solutions is probably due to a matter of convergence tolerance in the decision variables and/or objective function.

We agree with the reviewer that there is the possibility that a smarter mathematical formulation of the optimization problem exists. However, although the model is relatively simple, the optimization is done according to the position of each sampling point. Therefore, an experimental design with 18 sampling points has 18 decision variables. We believe that a deeper study of the optimization problem, although interesting from the point of view of optimization theory, would only have a small contribution to the research question (how the properties of the FIM and MC simulations can aid in experimental design) and would increase the complexity of the analysis.

- [Lines 167- 170]: E-criterion attempts to minimise the maximum uncertainty in parameter estimates (not the parameter with the highest error). In addition, the authors should clearly state which aspects of D-optimality meet ‘circumstances’ in their study case.

We acknowledge the reviewer’s comment. We have referred to the E-criterion in the text to make the reader aware of the fact that there are other alternatives. However, a variety of criteria have been suggested for the quantification of the information observable using an experimental design (A-criterion, C-criterion, D-criterion, E-criterion, T-criterion, G-criterion, I-criterion…) and many of them even have modified version (e.g. the A-criterion) with better properties from the point of view of the optimization problem. As well as with the previous comment, we believe that a comparison between the designs identified using each one of the criteria listed, although very interesting from the point of view of experimental design theory, would only bring a small contribution to the research question analysed. Hence, it is left for future work. In any case, the R package bioOED with which the calculations can be reproduced, allows the user to switch between D and modified E-criterion to explore other possible designs.

According to the reviewer’s remarks and the paragraph has been rephrased including additional information:

“A popular alternative is the E-criterion, which tries to minimize the maximum uncertainty in parameter estimates (in the case studied here, the uncertainty of the parameter with the highest error) (Balsa-Canto et al., 2008). Because this article tries to illustrate how computational methods can be used to aid experimental design and because this criterion has already been applied in a similar problem (Garre et al., 2018b), this study is limited to the results of the D-criterion. A comparison between the precision of different designs is left for future work.”

- Among the mentioned limitations of the FIM-based approach, local validity in the parameter space (i.e. computations are performed in the neighbourhood of the a priori unknown optimal/true parameters values) is the most relevant. I could not find any reference to how the initial uncertainty in parameter estimates was accounted for. This is a crucial point for applicability of the outlined methods. The application of the methods cannot precede the acquisition of some experimental data.

We acknowledge the reviewer’s comment. The need for nominal parameters values is a known limitation of the approach to OED based on the FIM. We have used model parameters taken from the literature for experimental conditions similar to the ones considered in our study. This approach has also been applied in other articles that also studied OED in the context of predictive microbiology (Akkermans et al., 2018b, 2018a; Bernaerts et al., 2000; Garre et al., 2018b; Poschet et al., 2005; Stamati et al., 2016; van Derlinden et al., 2010; Versyck et al., 1999, 1997). Due to the availability of extensive reviews and databases of microbial inactivation (Baranyi and Tamplin, 2004; Doyle et al., 2001; Doyle and Mazzotta, 2000; van Asselt and Zwietering, 2006), approximate values of the model parameters can be found for most conditions.

In a practical setting, the experimental design could be updated after the first repetition. This approach, quite common in, for instance, systems biology, is usually not required in predictive microbiology, due to the lower dimensionality of the models.

In any case we find the reviewer remark very interesting and we will consider performing a new study based on global sensitivity analysis to guide predictive microbiologist in the uncommon cases where nominal values for the parameters cannot be found anywhere.

- An elusive comparison of the informative content of thermal profiles is presented. Due to the low dimensionality of the mathematical model, the simultaneous optimisation of i) number of sampling times, ii) their location and iii) the perturbation profile would have been feasible and of high interest.

See response to the first major comment. We agree with the reviewer that finding optimal temperature profiles for designing optimal experiments or in other tasks like process design and optimization is a very relevant engineering question. However, as mentioned above, the temperature profile in this context is crucial for biological aspects like the microorganism acclimation. In such cases, the model that best describes the microbial counts within a thermal inactivation process would be different, with a different number of parameters. We have preferred to let the temperature profile a as discrete given factor (i.e., by considering 3 different profiles which could have been extended to other commonly used in research or industry) to illustrate the main objective of the paper: OED + MC simulations is an effective methodology to select the sampling points in thermal microbial inactivation and get the maximum information.

- The conclusions drawn from the research provide limited insights and a more in depth analysis should be performed. It is intuitive that experience-based experimental schemes provide a lower bound of the performances of OED, otherwise we would not put effort in optimisation. The fact that one perturbation scheme results more informative than others is useful only if patterns identified support the extrapolation of ‘rules for informativeness’. Finally it seems obvious that data are not equally informative, so that increasing their cardinality might not convey additional information in uniform schemes.

We acknowledge the reviewer’s comment. She raises several objection that we will respond one by one.

We agree that experience-based are a lower bound for OED. That’s why we always try to include uniform designs as a baseline for comparison with the OED (the definition of an “experience-based” one can be researcher-dependent). We have found some patterns of perturbation, shown in Figure 2, that are dependent on the temperature profile and are stable with the number of sampling points. However, we have not included the shape of the thermal profile in those patterns due to the biological arguments we have given in our response to the previous comments.

Regarding the uniform design, an expert on experiment design may find this point obvious. But we believe that researchers with a background that is more focused on microbiology expect that increasing the number of sampling points would reduce the uncertainty in parameter estimates. Indeed, it is very rare for articles in predictive microbiology to justify the number of sampling points. In this research work we clearly illustrate that increasing the number of sampling points does not ensure that the uncertainty in parameter estimates is reduced. We could not find such result published before in the context of predictive microbiology and we believe that some researchers may find this point, at least, surprising. We believe this is an interesting research finding, which can encourage predictive microbiologists with a background in microbiology to apply OEDs.

Our goal with this work was to illustrate how computational tools (the properties of the FIM and Monte Carlo simulations) can aid in experimental design. It can be used to compare between different designs (optimal/uniform, as well as between different profiles) and to evaluate how increasing the number of sampling points affects uncertainty. The results show that for each thermal profile, there is an upper bound for the amount of information that can be extracted, so uncertainty cannot be reduced below a minimum. This is already an interesting result for predictive microbiology, where it is usually assumed that uncertainty can be reduced to zero if enough information (experimental data, in this case) is gathered. Furthermore, we demonstrate that increasing the number of sampling points may actually increase uncertainty when a uniform sampling scheme is used. Finally, we illustrate how Monte Carlo simulations can be used to decide the number of sampling points in an experiment (maximum standard deviation of the parameters). To date, that was done based on previous experience, so there was a high risk of taking too many or too few points.

We believe that these conclusions are relevant for predictive microbiology and that have enough merit to be published.

**Minor comments**

We thank the reviewer for reporting the list of found typos and errors. Every minor correction has been applied as suggested. Furthermore, the whole manuscript has been proof-read and several typos have been corrected.

- [Lines 458-461]: Unclear, rephrase the sentence

The sentence has been rephrased as:

“Also, some typical model assumptions (e.g. homogeneity of the residuals) can be checked by means of the Monte Carlo simulations”

**References**

Akkermans, S., Logist, F., Van Impe, J.F., 2018a. Parameter estimations in predictive microbiology: Statistically sound modelling of the microbial growth rate. Food Res. Int. 106, 1105–1113. https://doi.org/10.1016/j.foodres.2017.11.083

Akkermans, S., Nimmegeers, P., Van Impe, J.F., 2018b. Comparing design of experiments and optimal experimental design techniques for modelling the microbial growth rate under static environmental conditions. Food Microbiol. https://doi.org/10.1016/j.fm.2018.05.010

Balsa-Canto, E., Alonso, A.A., Banga, J.R., 2008. Computational procedures for optimal experimental design in biological systems. IET Syst. Biol. 2, 163–172. https://doi.org/10.1049/iet-syb:20070069

Baranyi, J., Tamplin, M.L., 2004. ComBase: A Common Database on Microbial Responses to Food Environments. J. Food Prot. 67, 1967–1971. https://doi.org/10.4315/0362-028X-67.9.1967

Bernaerts, K., Versyck, K.J., Van Impe, J.F., 2000. On the design of optimal dynamic experiments for parameter estimation of a Ratkowsky-type growth kinetics at suboptimal temperatures. Int. J. Food Microbiol. 54, 27–38.

Conesa, R., Andreu, S., Fernández, P.S., Esnoz, A., Palop, A., 2009. Nonisothermal heat resistance determinations with the thermoresistometer Mastia. J. Appl. Microbiol. 107, 506–513. https://doi.org/10.1111/j.1365-2672.2009.04236.x

Corradini, M.G., Peleg, M., 2009. Dynamic Model of Heat Inactivation Kinetics for Bacterial Adaptation. Appl. Environ. Microbiol. 75, 2590–2597. https://doi.org/10.1128/AEM.02167-08

Corradini, M.G., Peleg, M., 2004. Demonstration of the applicability of the Weibull–log-logistic survival model to the isothermal and nonisothermal inactivation of Escherichia coli K-12 MG1655. J. Food Prot. 67, 2617–2621.

de Jong, A.E.I., van Asselt, E.D., Zwietering, M.H., Nauta, M.J., de Jonge, R., 2012. Extreme Heat Resistance of Food Borne Pathogens Campylobacter jejuni, Escherichia coli, and Salmonella typhimurium on Chicken Breast Fillet during Cooking [WWW Document]. Int. J. Microbiol. https://doi.org/10.1155/2012/196841

de Jonge, R., 2019. Predictable and unpredictable survival of foodborne pathogens during non-isothermal heating. Int. J. Food Microbiol. 291, 151–160. https://doi.org/10.1016/j.ijfoodmicro.2018.11.018

Dolan, K.D., Valdramidis, V.P., Mishra, D.K., 2013. Parameter estimation for dynamic microbial inactivation: which model, which precision? Food Control, Predictive Modelling of Food Quality and Safety 29, 401–408. https://doi.org/10.1016/j.foodcont.2012.05.042

Doyle, M.E., Mazzotta, A.S., 2000. Review of Studies on the Thermal Resistance of Salmonellae. J. Food Prot. 63, 779–795. https://doi.org/10.4315/0362-028X-63.6.779

Doyle, M.E., Mazzotta, A.S., Wang, T., Wiseman, D.W., Scott, V.N., 2001. Heat Resistance of Listeria monocytogenes. J. Food Prot. 64, 410–429. https://doi.org/10.4315/0362-028X-64.3.410

Franco-Vega, A., Ramírez-Corona, N., López-Malo, A., Palou, E., 2015. Estimation of Listeria monocytogenes survival during thermoultrasonic treatments in non-isothermal conditions: Effect of ultrasound on temperature and survival profiles. Food Microbiol. 52, 124–130. https://doi.org/10.1016/j.fm.2015.07.006

Garre, A., Egea, J.A., Iguaz, A., Palop, A., Fernandez, P.S., 2018a. Relevance of the Induced Stress Resistance When Identifying the Critical Microorganism for Microbial Risk Assessment. Front. Microbiol. 9. https://doi.org/10.3389/fmicb.2018.01663

Garre, A., González-Tejedor, G., Peñalver-Soto, J.L., Fernández, P.S., Egea, J.A., 2018b. Optimal characterization of thermal microbial inactivation simulating non-isothermal processes. Food Res. Int. 107, 267–274. https://doi.org/10.1016/j.foodres.2018.02.040

Garre, A., Huertas, J.P., González-Tejedor, G.A., Fernández, P.S., Egea, J.A., Palop, A., Esnoz, A., 2018c. Mathematical quantification of the induced stress resistance of microbial populations during non-isothermal stresses. Int. J. Food Microbiol. 266, 133–141. https://doi.org/10.1016/j.ijfoodmicro.2017.11.023

Haberbeck, L.U., Dannenhauer, C., Salomão, B.D.C.M., De Aragão, G.M.F., 2013. Estimation of the Thermochemical Nonisothermal Inactivation Behavior of Bacillus Coagulans Spores in Nutrient Broth with Oregano Essential Oil. J. Food Process. Preserv. 37, 962–969. https://doi.org/10.1111/j.1745-4549.2012.00745.x

Hassani, M., Cebrián, G., Mañas, P., Condón, S., Pagán, R., 2006. Induced thermotolerance under nonisothermal treatments of a heat sensitive and a resistant strain of Staphylococcus aureus in media of different pH. Lett. Appl. Microbiol. 43, 619–624. https://doi.org/10.1111/j.1472-765X.2006.02014.x

Hassani, M., Manas, P., Pagán, R., Condón, S., 2007. Effect of a previous heat shock on the thermal resistance of Listeria monocytogenes and Pseudomonas aeruginosa at different pHs. Int. J. Food Microbiol. 116, 228–238.

Huang, L., 2013. Determination of thermal inactivation kinetics of Listeria monocytogenes in chicken meats by isothermal and dynamic methods. Food Control 33, 484–488. https://doi.org/10.1016/j.foodcont.2013.03.049

Huertas, J.-P., Aznar, A., Esnoz, A., Fernández, P.S., Iguaz, A., Periago, P.M., Palop, A., 2016. High Heating Rates Affect Greatly the Inactivation Rate of Escherichia coli. Front. Microbiol. 7. https://doi.org/10.3389/fmicb.2016.01256

Huertas, J.-P., Ros-Chumillas, M., Esteban, M.-D., Esnoz, A., Palop, A., 2015. Determination of Thermal Inactivation Kinetics by the Multipoint Method in a Pilot Plant Tubular Heat Exchanger. Food Bioprocess Technol. 8, 1543–1551. https://doi.org/10.1007/s11947-015-1525-9

Mattick, K.L., Legan, J.D., Humphrey, T.J., Peleg, M., 2001. Calculating Salmonella inactivation in nonisothermal heat treatments from isothermal nonlinear survival curves. J. Food Prot. 64, 606–613.

Poschet, F., Geeraerd, A.H., Van Loey, A.M., Hendrickx, M.E., Van Impe, J.F., 2005. Assessing the optimal experiment setup for first order kinetic studies by Monte Carlo analysis. Food Control 16, 873–882. https://doi.org/10.1016/j.foodcont.2004.07.009

Ros-Chumillas, M., Esteban, M.-D., Huertas, J.-P., Palop, A., 2015. Effect of Nisin and Thermal Treatments on the Heat Resistance of Clostridium sporogenes Spores. J. Food Prot. 78, 2019–2023.

Stamati, I., Akkermans, S., Logist, F., Noriega, E., Van Impe, J., 2016. Optimal experimental design for discriminating between microbial growth models as function of suboptimal temperature: From in silico to in vivo. Food Res. Int. 89, 689–700. https://doi.org/10.1016/j.foodres.2016.08.001

Stasiewicz, M.J., Marks, B.P., Orta-Ramirez, A., Smith, D.M., 2008. Modeling the Effect of Prior Sublethal Thermal History on the Thermal Inactivation Rate of Salmonella in Ground Turkey. J. Food Prot. 71, 279–285. https://doi.org/10.4315/0362-028X-71.2.279

Valdramidis, V.P., Geeraerd, A.H., Bernaerts, K., Van Impe, J.F.M., 2008. Identification of non-linear microbial inactivation kinetics under dynamic conditions. Int. J. Food Microbiol. 128, 146–152.

Valdramidis, V.P., Geeraerd, A.H., Van Impe, J.F., 2007. Stress-adaptive responses by heat under the microscope of predictive microbiology: Modelling the microbial heat resistance. J. Appl. Microbiol. 103, 1922–1930. https://doi.org/10.1111/j.1365-2672.2007.03426.x

van Asselt, E.D., Zwietering, M.H., 2006. A systematic approach to determine global thermal inactivation parameters for various food pathogens. Int. J. Food Microbiol. 107, 73–82. https://doi.org/10.1016/j.ijfoodmicro.2005.08.014

van Derlinden, E., Balsa-Canto, Van Impe, 2010. (Optimal) experiment design for microbial inactivation, in: Progress on Quantitative Approaches of Thermal Food Processing - Valdramidis, V.P. & Van Impe, J.F.M. (Eds), Advances in Food Safety and Food Microbiology. Nova Publishers, pp. 67–98.

Versyck, K.J., Bernaerts, K., Geeraerd, A.H., Van Impe, J.F., 1999. Introducing optimal experimental design in predictive modeling: A motivating example. Int. J. Food Microbiol. 51, 39–51. https://doi.org/10.1016/S0168-1605(99)00093-8

Versyck, K.J., Claes, J.E., Impe, J.F.V., 1997. Practical Identification of Unstructured Growth Kinetics by Application of Optimal Experimental Design. Biotechnol. Prog. 13, 524–531. https://doi.org/10.1021/bp970080j
